# Do Playgrounds Help Develop Children’s Fundamental Movement Skills? Using Direct Video Observations to Investigate

**DOI:** 10.3390/sports13090289

**Published:** 2025-08-27

**Authors:** Amy Stringer, Ruth D. Postlethwaite, Matteo Crotti, Michael Duncan

**Affiliations:** 1 Centre for Physical Activity, Sport and Exercise Sciences, Coventry University, Coventry CV1 5FB, UK; stringer3@uni.coventry.ac.uk (A.S.); postlethwr@uni.coventry.ac.uk (R.D.P.); 2 Department of Human and Social Science, University of Bergamo, 24127 Bergamo, Italy; matteo.crotti@unibg.it

**Keywords:** parks, Observational System for Recording Physical Activity in Children (OSRAC), camera assisted, physical literacy, physical activity, playground equipment, playground features

## Abstract

Playgrounds are global environments that are purpose made for children and can offer a variety of opportunities for children to be physically active and practice their fundamental movement skills (FMS), which can lead to future physical activity and sport participation. Previous research highlighted that children engage in different types of physical activity (PA) depending on playgrounds apparatus and area. However, there is a paucity of research that investigates the link between playground features, structures, PA, and FMS. This study sought to assess the impact of different playgrounds on PA type PA intensity and the types of FMS completed. This observational study examined 29 (M = 10, F = 19) children’s behaviours on three different playgrounds. Video cameras were placed strategically across the three playgrounds to allow for footage to be captured and analysed using the Observational System for Recording Physical Activity in Children (OSRAC). One-way ANOVA was used to examine the different OSRAC categories across the three playgrounds. Climbing equipment (average 1217.10 s) was the frequently used type of apparatus, standing was the most commonly performed type of activity (average 377.60 s) and stationary movements whilst moving limbs were the most regularly (average 605.13 s) performed type of PA intensity. There were no instances of any throwing, catching, or kicking activities performed across the three playgrounds. Results suggest that public playgrounds do not facilitate more intense types of PA, nor object control skills due to a lack of suitable equipment.

## 1. Introduction

Physical activity (PA) is an important factor that contributes to the development, health and wellbeing of children [1,2,3,4]. PA positively impacts cognitive development in terms of creativity, problem solving, and memory [5,6,7]. Likewise, PA also impacts physical health by improving strength, flexibility, and cardiovascular ability, therefore reducing the impact of obesity [8,9,10,11]. PA engagement has also been shown to influence the development of fundamental movement skills (FMS) in children [12,13,14], which are central to future physical activity and sport participation. FMS, considered to be the building blocks of movement and associated with lifelong PA engagement, can be divided in three categories: locomotor (e.g., walking, running, jumping), stability skills (e.g., balancing, standing), and object control skills (e.g., throwing, catching, kicking) [15,16,17]. These FMS underpin everyday PA as well as more sports-based contexts [10,13,14]. In recognition of the benefits of FMS and PA, the World Health Organisation (WHO) recommends that children should engage in a minimum of 60 min of moderate-to-intense PA every day [1,18]. Despite this, the PA levels and FMS abilities of children are repeatedly reported to be low worldwide [19,20]. The WHO has suggested that the outdoor environment in which children engage in can play a significant part in increasing PA and potentially FMS [1,18]. This is further supported by the Ecological Dynamics Theory which states that the environment that children interact with impacts skill acquisition, such as FMS, which in turn impacts PA engagement and play [20,21].

One outdoor space that is designed and built specifically for children are playgrounds [21,22]. Playgrounds are present across the world and can offer a variety of opportunities for children to be physically active and practice FMS [23]. Amholt et al. [24] utilised GPS and accelerometers to investigate the PA of children (*n* = 376) on four different playgrounds and reported that soccer fields and areas with equipment resulted in higher PA overall, whilst climbing equipment in particular facilitated greater PA in girls. Andersen et al. [25] utilised GPS and accelerometers to investigate the PA of children (*n* = 509) on playgrounds and reported that the ‘solid surface’ areas resulted in the greatest time (47%) spent in sedentary activity whilst the ‘grass’ areas resulted in the greatest moderate-to-vigorous physical activity (MVPA) (27%), followed closely by the ‘playground’ areas (26%). These findings differed between genders, however, as girls were reported to accumulate more sedentary activity in all areas of the playgrounds when compared to boys. Similar results were reported by Nielsen et al. [26], who investigated the PA of 417 children in relation to the play facilities and physical structures available at seven schools. The results reported that the number of physical structures was positively associated with PA and each additional structure resulted in 4 min (*p* = 0.001) more MVPA. Cohen et al. [27] 2020, conducted a large investigation of 162 US playgrounds and reported that children (*n* = 150) and adults (*n* = 125) were more likely to visit playgrounds than teenagers (*n* = 23) and seniors (*n* = 2). Additionally, males (54.0%) were observed engaging in more moderate-to-vigorous intensity PA than females (45.8%). However, the findings of Cohen et al. [27] do not consider the impact of variables such as body mass index (BMI), which further literature has highlighted may impact playground behaviors [28,29]. It is important to note this point around BMI, which often is ignored in studies investigating playground activity. As engagement on playgrounds requires carrying of body mass in space, i.e., through travelling on/through the play space, climbing, crawling, and other physical activities, body shape will likely influence a child’s interaction with the play environment. Thus, BMI is a likely confounder that needs to be accounted for when exploring how children undertake PA in such environments. These findings highlight that playgrounds can be opportune environments for children to be active and that different playgrounds features and structures impact the type and intensity of PA engaged in.

Whilst there is a plethora of research investigating playgrounds and PA, there is limited research investigating playgrounds and FMS [23]. Pawlowski et al. [30] recently conducted a scoping review and found only 14 articles that examined FMS in relation to playgrounds. Of the 14 articles, four were conducted in public playgrounds, seven were conducted in early childhood education centres, two in rehabilitation centres, and one in a primary school. Early childhood education centres and primary schools often have limited space available for playgrounds and equipment. Likewise, they cater to a limited age group, and the equipment available reflects that age group. Rehabilitation playgrounds are often designed to support recovery and have specialised equipment as a result. Therefore, the results of findings from playgrounds in these environments cannot be easily generalised due to specification in playground design that cater to specific populations. In contrast, research conducted on public or neighbourhood spaces is more easily applied as these environments are more widespread and must cater to a wider population (e.g., equipment to suit a wider age range, must be accessible to a larger number of children at one time, etc.). However, as Pawlowski et al. [30] highlighted, only four articles examined public playgrounds and FMS. This shows that even within the limited amount of playground and FMS research, there is a gap concerning public playgrounds and FMS.

Two of the four articles identified by Pawlowski et al. [30] reported that FMS ability was improved through structured and semi-structured play when compared to unstructured play [31,32]. A third article reported that 53.7% of parents agreed that playgrounds had a positive impact on their child’s FMS [33]. Whilst each of these articles addresses the gap in the literature, they do not provide insight into the manner in which children interact with the playground and the type of PA that they engage in. The context as to how and why FMS are improved or supported by playgrounds is missing. The only article, to date, that investigates public playgrounds and FMS whilst offering information how children interact with playgrounds is Adams et al. [34].

Adams et al. [34] investigated how children interacted with three different types of playgrounds (traditional, adventure, and contemporary) in Australia and the FMS that the children engaged in. A combined use of accelerometers (body-worn devices to measure PA intensity) and direct observations (systematic scanning of an area and recording the activity that takes place) were used to understand the PA in the different playgrounds. The results revealed that children performed locomotor skills (31.3%) more frequently than body management (15.2%) and object control skills (0.6%), and that a wider variety of equipment was utilised on the contemporary and adventure playgrounds. However, there were no statistically significant differences between the playgrounds for the type of PA or FMS performed. The authors concluded that the lack of statistical findings may be due to the low overall FMS of Australian children. However, the authors did not test the FMS abilities of the children in their sample so the claims cannot be substantiated. The results may also be impacted by the methods utilised as direct observations have been known to overestimate and underestimate PA [35]. Whilst the work of Adams et al. (2018) [34] is able to address the gap in FMS and playground research, there is still some ambiguity surrounding the results.

To address the gaps in the literature, whilst building upon the strengths of research previously conducted, the aims of this study were to use video-assisted direct observations to understand how children interact with different types of public playground in terms of movement behaviours and identify if their FMS capability had an impact. This study sought to assess the impact of different playgrounds on PA type and intensity whilst accounting for the potential impact of BMI, FMS ability (TGMD (Test of Gross Motor Development) score), gender, and age.

## 2. Materials and Methods

### 2.1. Setting

**Figure A1:**
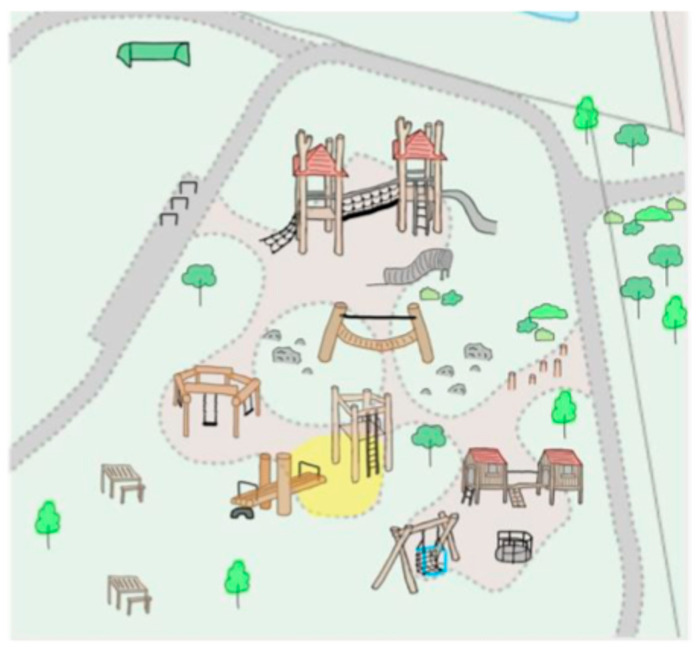
Depiction of the equipment at the first playground.

**Figure A2:**
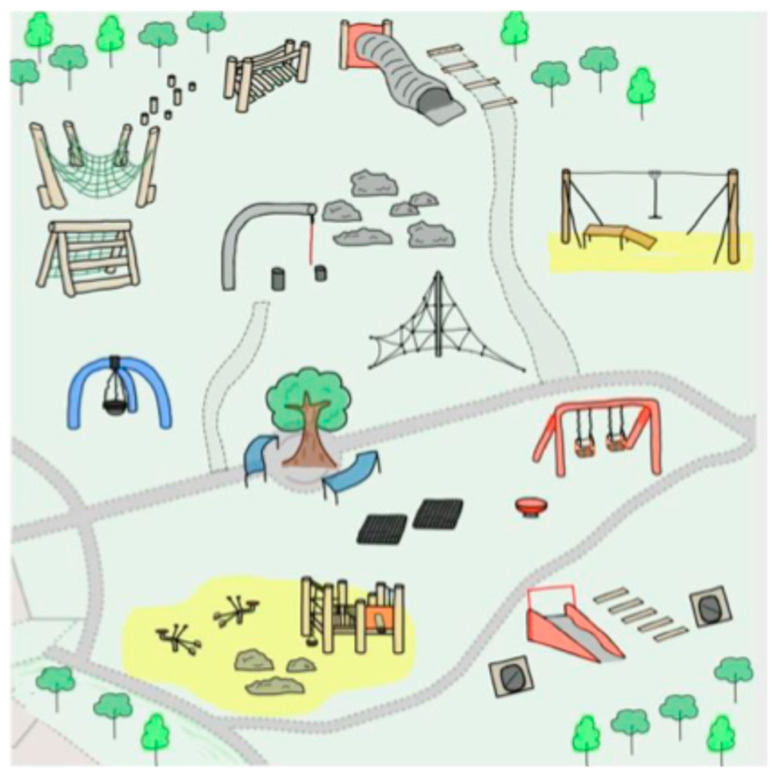
Depiction of the equipment at the second playground.

**Figure A3:**
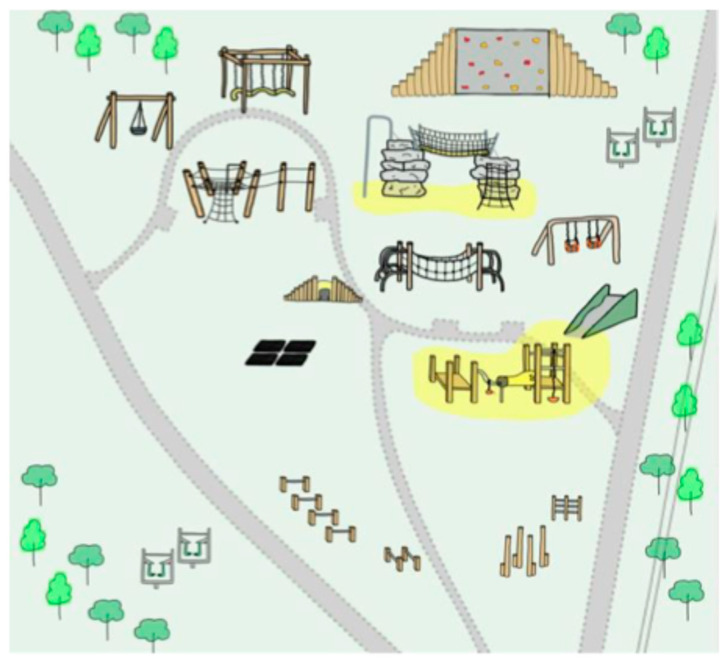
Depiction of the equipment at the third playground.

Three playgrounds located within 7 miles (11 km) of the school that the participants attended were selected based on their size (primarily the ability to accommodate 30 or more children at one time) and their varying equipment, layout, and design. Each of the playgrounds were of similar size (playground 1 = 4689.52 m^2^, playground 2 = 4940.72 m^2^, playground 3 = 4917.73 m^2^) and contained somewhat similar equipment (e.g., climbing frame, slides), though there were some original features and equipment (e.g., giant bridge, gym equipment, zip line) present on each playground (see Appendix A Figure A1, Figure A2 and Figure A3 for playground depictions).

### 2.2. Participant Recruitment

Participants (M = 10, F = 19, Caucasian = 55%) aged 5 to 10 years old were recruited from a community (Index of multiple deprivation decile score for this postcode area = 7) in Rugby, UK, to participate in the study. This community was chosen due to its proximity to the playgrounds in this study. Flyers were distributed via the local community social media pages to recruit participants. This study was limited to recruiting 30 participating children due to UK childcare guidance and laws that dictate adult-to-child ratios, and because there were a limited number of adults available to assist [36]. Children of mixed genders and of various ages were recruited to ensure heterogeneity and allow for variations in children’s abilities, PA level, and play preferences. This decision mimics the use of public playgrounds in community settings and thus ensured ecological validity. Of the 30 children recruited, 1 withdrew (no reason given for withdrawal) resulting in 29 participants total. Complete data were available for all three playgrounds for 26 children due to attrition in which 3 participants did not attend all three playgrounds. Playground 1 had 29 participants, playground 2 had 28 participants, and playground 3 had 26 participants (Tables 2 and 3 highlight the number of participants present during data collection on each playground in the caption).

### 2.3. Ethics

Ethical approval was granted by the Institutional Review Board (or Ethics Committee) of Coventry University (project 136524). Consent was gained from the participants and their parent/s guardians prior to data collection. Following data collection all participant information was anonymized for analysis. Data collection took place on three public playgrounds; therefore, members of the general public were informed that the use of cameras and video recording would be taking place. This was achieved by the research team placing notices around the playground and verbally informing individuals who were present at the playgrounds.

### 2.4. Data Collection

Data collection occurred from the 23 to 25 August 2023 during the UK school summer holiday period. One playground was visited on each day of the data collection period, and the weather was similar on all three days (sunny, with temperatures ranging from 18 to 22 degrees Celsius (°C)). Each playground was visited for a 60 min period in the morning (to avoid the worst of the sun and summer heat). The study used four different data collection methods, which are outlined below.

### 2.5. Test of Gross Motor Development-3 and Anthropometric Measurements

The height (cm) and body mass (kg) of the participants were recorded using a stadiometer (Seca 213 portable stadiometer, Hamburg, Germany) and scales (Seca 875 electronic class 3 scales, Hamburg, Germany). These measurements were used to calculate age-adjusted BMI (mean ± SD = 16.64 ± 2.19, 6 children overweight, 23 normal weight) [37]. The Test of Gross Motor Development-3 (TGMD) was used to measure children’s gross motor skills (mean ± SD = 86.09 ± 9.15). Following demonstration and verbal description of each movement, children were recorded performing each of the skills twice. Analysis of each skill against the performance criteria was completed post-data collection.

### 2.6. Video Footage Collection

Eight cameras (Panasonic HC-V380EB-K Full-HD Handheld Video, Osaka, Japan) on tripods were set strategically across each playground. This ensured that each piece of equipment was within view of a camera. Some pieces of equipment required two cameras to observe activity on all sides because they were so large. Cameras were initialised so that all cameras and footage could be synchronised to enable easier analysis of the footage post-recording.

To overcome the Hawthorne effect and record data that was as true to real life as possible, only video footage from the middle 30 min of the hour at each playground was used [38]. The first 15 and final 15 min of video footage were removed so that external factors that may have influenced behaviours (e.g., becoming familiar with the camera being present, recovering from transport to the playground and gathering together to leave the playground) were not collected. Therefore, each playground had a total of 30 min of data recorded across eight cameras, resulting in 240 min (4 h) worth of footage for each playground and 720 min (12 h) of footage for all three playgrounds. Multiplied by the number of children present on each playground (playground 1 = 29 participants, playground 2 = 28 participants and playground 3 = 26 participants) results in 19,920 min (332 h) total footage analysed.

Footage was streamed through NacSport © Scout Version 9.1.2. A tagging window was created that allowed for the Observational System for Recording physical Activity in Children (OSRAC) to be computerised. A tagging window is a visual representation of the information that is being collected, and buttons are used to select or deselect when time data is collected or not, against the video footage. For example, when a child is running on the video footage, the button for running is selected, and when the child stops running, the button is deselected. The selection and deselection of button generates accurate time-based data.

For the purposes of this study, each playground has a unique tagging window created where the OSRAC categories were used to identify the type of PA children were engaging in and the intensity of that activity, in relation to the different pieces of equipment present on each playground.

### 2.7. Observational System for Recording Physical Activity in Children (OSRAC)

OSRAC was employed to determine children’s movement behaviours and was chosen over other observational approaches because of the depth of information that it can provide in children’s PA and interactions [39,40,41,42]. A modified and computerised version of the OSRAC was used to measure the dwell time (i.e., time spent on each piece of equipment/area of the playground), physical activity type, and physical activity intensity of each participant on each of the three playgrounds. The 332 h of footage were rewatched for each of the 29 participants using OSRAC to understand their PA. Rather than using momentary time sampling, where a researcher observes a child for 5 s every 25 s, the behaviour of each child was recorded continually for the entire 30 min period they were recorded on each playground. This resulted in second-by-second, time-based data being produced, rather than frequency of activity being produced. For example, if a child was climbing on a climbing frame, then this activity would be recorded for its entire duration, until the child changed activity (e.g., stopped climbing and started walking), or moved onto another piece of equipment (e.g., a slide attached to the climbing frame). Therefore, this study utilised the OSRAC to collect nomothetic, multidimensional and inter-sessional data [40].

In addition to the activities identified in the OSRAC, “Transitioning” and “Chasing games” were added as activity types. This was performed following pilot testing and previous research that highlighted that these types of activity could be important; therefore, we wanted to capture these elements as metrics [20,34,43]. “Transitioning” was used in the previous literature by Foweather et al. (2021) [43] and for the purposes of this study was defined as instances where children were “in spaces between pieces of equipment or moving directly from one piece of equipment to another”. To clarify, transitioning is not a type of PA, such as walking or running, and is classified as a lack of equipment. “Chasing games” were defined as instances where children were “engaging in chasing games, such as tag or “chasey”, where the objective was to ‘catch’ another child or children”. Whilst “chasing games” is not a type of PA included in OSRAC it was incorporated into this study due to the findings of Adams et al. (2018) [34]. The authors highlighted that participants in their study were frequently observed taking part in chasing games; however, they did not measure it in their study so could not make explicit conclusions regarding chasing games. “Chasing games” has been used and validated in other direct observation approaches, such as the System for Observing Play and Recreation in Communities (SOPARC) [44,45].

### 2.8. Reliability

The main researcher went through a period of training, with an expert, to use the OSRAC tool and complete the TGMD assessment. During this period 100% inter-rater reliability was scored for both the OSRAC tool and TGMD assessment. An initial trial of the tagging window was conducted to assess suitability and accuracy prior to data collection. As a result of the multiple camera angles utilised, there was only one observer, as multiple observers were not required. In instances of uncertainty accuracy was checked by an expert.

### 2.9. Data Analysis

Using the various OSRAC categories, data was collected on where the participants were located on each playground (termed dwell time) and what type and intensity of PA they were performing. To explore what types of activity were undertaken across the three playgrounds, descriptive data were calculated for each of the OSRAC categories on each of the playgrounds

To examine any differences in PA types and PA intensity on each playground, a series of one-way repeated measures analysis of variance (ANOVA) was conducted with the various OSRAC categories across the three different playgrounds as the dependent variable and gender as the between-subjects factor. Recognising the age spread of the participants, and that weight status and motor competence might confound results data were subsequently rerun using a series of repeated measures analysis of covariance (ANCOVA) with various OSRAC categories across the three different playgrounds as the dependent variable, and gender as the between subjects factor controlling for age, BMI, and TGMD scores (complete data for TGMD scores were available for 22 children).

Where any significant differences were identified, Bonferroni post hoc pairwise comparisons were used to examine where the differences lay. Partial eta^2^ (Pη^2^) was used as a measure of effect size. Where a covariate was significant, parameter estimates (beta weights) were used to understand the association between the covariate and the dependent variable. The statistical package for social sciences (IBM SPSS version 30) was used for all analyses.

## 3. Results

### 3.1. Equipment Dwell Time

Each of the three playgrounds in this study had unique features and layouts; therefore, direct comparisons between each piece of equipment could not be made, hence no statistical test was carried out. However, descriptive statistics for the general types of equipment grouped together across all three playgrounds were calculated (see Table 1).

On average, children were observed on the climbing equipment more frequently (1217.10 s) than on any other type of equipment. However, the swing equipment (828.71 s), sand equipment (746.70 s), seated equipment (728.59 s), and transitioning (721.28 s) were interacted with more frequently than activity trail equipment (214.90 s) and the other equipment (243.69 s). There were some differences in gender according to the different equipment types. Males spent notably more time on the activity trail equipment (258.13 s), sand equipment (1241.30 s) and transitioning (820.50 s), whilst females spent notably more time on the climbing equipment (1278.37 s), seated equipment (746.42 s) and swing equipment (875.68 s). We note that there are occasions where the mean for one sex is substantially greater or lower than the overall mean for that dwell type. For example, the mean for sand play is 746.7 s compared to a mean dwell time for sand play in males of 1241 s and 419 s for females. While this may appear counterintuitive, the variability in such data should be acknowledged, whereby there was considerable variation in sand play for males (SD = 1124 s) and the clear distinction in dwell time in sand play between males and females, resulting in the mean for the group being intermediate between the males and females.

### 3.2. Physical Activity Type

The types of PA that children engaged in on the playground varied, but some types of PA were performed more frequently than others (see Table 2). Across the three playgrounds, standing (377.60 s) was the most frequently performed PA type, followed by walking (267.82 s), sand play (260.38 s), swinging (224.70 s), and sitting/squatting (222.29 s). The least frequently performed PA types were crawling (12.11 s), jumping/skipping (17.11 s), and sliding (17.84 s).

On the first playground, standing (348.38 s) was the most frequently performed PA type, followed by swinging (298.10) and walking (261.28 s). There were some differences between gender as males spent notably more time performing pushing/pulling activities (149.86 s vs. 67.64 s), running (378.40 s vs. 73.84 s), sand play (378.40 s vs. 101.67 s) and standing (436.90 s vs. 301.79 s). In contrast, females spent somewhat more time performing sitting/squatting (208.21 s vs. 178.00 s), swinging (306.53 s vs. 272.80 s) and walking (270.37 s vs. 244.00 s).

On the second playground, the most frequently observed types of PA children engaged in overall were standing (400.04 s), walking (255.04 s), and sitting/squatting (217.35 s). This was followed by sand play (140.00 s), climbing/hanging (116.89 s), lying down (88.00 s), and pulling/pushing (81.19 s). The other types of PA were performed notably less frequently (61.11 s to 9.57 s). Again, there were some differences between genders as males performed more crawling (48.33 s vs. 4.00 s), rough and tumble (61.00 s vs. 11.00 s), and sand play (242.57 s vs. 56.56 s) than females. Females performed more lying down (182.50 s vs. 50.20 s) and sitting/squatting (256.61 s vs. 156.00 s) than males.

On the third playground, the most frequently observed type of PA was sand play (439.63 s) followed by standing (386.04 s), walking (288.88 s), sitting/squatting (254.96 s), and climbing hanging (246.50 s). Somewhat frequently performed were pushing/pulling (187.29 s), chasing games (172.00 s), running (120.77 s), and rough and tumble (91.17 s). Crawling (9.27 s), jumping/skipping (20.39 s), lying down (50.00 s), and sliding (27.08 s) were performed the least frequently. Males performed a greater amount of pushing/pulling (347.67 s vs. 99.82 s), running (142.50 s vs. 107.19 s), and sand play (576.67 s vs. 376.38 s) PA types. Females performed more rough and tumble (127.00 s vs. 73.25 s), sitting/squatting (298.25 s vs. 185.70 s), standing (411.31 s vs. 345.60 s), and walking (326.13 s vs. 229.30 s).

### 3.3. Physical Activity Intensity

The most frequently performed PA intensity was limbs (605.13 s) (i.e., standing whilst moving arms or legs), followed by moderate intensity PA (572.69 s), slow/easy intensity PA (368.78 s), stationary PA (43.86 s), and finally fast PA (26.31 s) (see Table 3).

Table 3 also shows that the intensities of PA children engaged in on the first playground, slow/easy (607.90 s), limbs (542.90 s), and moderate (316.76 s), were performed most frequently. There were also some differences between genders as males performed more limbs (586.00 s vs. 520.21 s), moderate (378.20 s vs. 284.42 s), and fast (58.70 s vs. 31.83 s) intensity activity than females, whilst females performed more slow/easy (613.63 s vs. 597.00 s) intensity activity.

The PA intensity that children most frequently performed overall on the second playground was limbs (627.54 s) (i.e., standing whilst moving arms and/or legs). This was followed by slow/easy (324.64 s) intensity activity and moderate (282.54 s) intensity activity, whilst stationary (31.65 s) and fast (29.95 s) intensity PA was performed the least frequently. There were some gender differences as males performed notably more stationary (58.14 s vs. 12.00 s), slow/easy (360.00 s vs. 290.67 s), and moderate (365.44 s vs. 237.83 s) intensity PA, whilst females performed more limbs (685.06 s vs. 555.00 s) activities.

The intensity of PA most frequently performed on the third playground was slow/easy (800.54 s) intensity, followed by limbs (650.81 s) and moderate (519.69 s) intensity PA. Fast (58.24 s) and stationary (44.67 s) were performed the least. Males performed notably more stationary (66.00 s vs. 2.00 s) and moderate (586.80 s vs. 477.75 s) intensity PA, whilst females performed more limbs (729.63 s vs. 524.70 s) and slow/easy (818.56 s vs. 771.71 s) intensity PA.

### 3.4. Playground 1

The results from one-way ANOVA indicated there were no significant interactions between any variables (all *p* > 0.05). One-way ANOVA determined that there were significant main effects for chasing games (F(1,24) = 7.1, *p* = 0.014)) and swinging (F(1,24) = 5.91, *p* = 0.023)). Bonferroni post hoc multiple comparisons showed that there was a significant difference between males and females for chasing games (*p* = 0.014, ES = 0.23) and swinging (*p* = 0.023, ES = 0.2). Comparing estimated marginal means for chasing games showed that males (mean = 82.01 s) performed more chasing games than females (mean = 20.84 s). Comparing estimated marginal means for swinging showed that females (mean = 263.19 s) performed more swinging than males (mean = 96.14 s).

The covariate ‘age’ was found to be significantly associated with chasing games (*p* = 0.001, β = 24.45 s, ES = 0.35), sitting/squatting (*p* = 0.029, β = −30.35 s, ES = 0.18), sliding (*p* = 0.012, β = 4.59 s, ES = 0.24), and swinging (*p* = 0.033, β = 45.68 s, ES = 0.18). This indicated that increasing age was associated with greater time in chasing games and sliding, whilst decreasing age was associated with sitting/squatting. The covariate TGMD score was associated with jumping/skipping (*p* = 0.032, β = 0.21, ES = 0.18) and swinging (*p* = 0.014, β = 2.48, ES = 0.23). This indicated that a higher TGMD score was associated with greater time spent in jumping/skipping and swinging.

### 3.5. Playground 2

One-way ANOVA determined that there were significant main effects for sand play (F(1,24) = 6.28, *p* = 0.019)), sitting/squatting (F(1,24) = 4.35, *p* = 0.048)), limbs (F(1,24) = 6.05, *p* = 0.022)), moderate (F(1,24) = 7.89, *p* = 0.01)), and stationary (F(1,24) = 6.73, *p* = 0.016)). Bonferroni post hoc multiple comparisons showed that there was a significant difference between males and females for sand play (*p* = 0.019, ES = 0.2), sitting/squatting (*p* = 0.048, ES = 0.15), limbs (*p* = 0.022, ES = 0.2), moderate (*p* = 0.01, ES = 0.25), and stationary (*p* = 0.016, ES = 0.22). Comparing estimated marginal means showed that males (mean = 196.76 s) performed more sand play than females (mean = 21.70 s), and males (mean = 356.17 s) performed more moderate intensity activity than females (mean = 228.91 s). Comparing estimated marginal means also showed that females (mean = 250.38 s) performed more sitting/squatting than males (mean = 132.77 s) and that females (mean = 672.35 s) performed more limbs intensity activity than males (mean = 478.64 s).

The covariate ‘age’ was found to be significantly associated with sliding (*p* = 0.001, β = −3.55 s, ES = 0.38) and fast intensity activity (*p* = 0.008, β = 7.13 s, ES = 0.26). This indicated that decreasing age was associated with greater sliding, whilst increasing age was associated with fast intensity activity. The covariate BMI was significantly associated with standing (*p* = 0.037, β = −39.80 s, ES = 0.17). This indicated that increasing BMI was associated with a decrease in standing. The covariate ‘TGMD’ was with limbs (*p* = 0.01, β = 2.97 s, ES = 0.24), indicating that greater TGMD was associated with greater limbs intensity activity.

### 3.6. Playground 3

One-way ANOVA determined that there were significant main effects for stationary (F(1,24) = 5.28, *p* = 0.031)). Bonferroni post hoc multiple comparisons showed that there was a significant difference between males and females for stationary (*p* = 0.031, ES = 0.18). Comparing estimated marginal means showed that males (mean = 15.72 s) performed more stationary intensity activities than females (mean = −1.22 s)

The covariate ‘age’ was found to be significantly associated with chasing games (*p* = 0.002, β = 26.47 s, ES = 0.33), rough and tumble (*p* = 0.002, β = 16.90 s, ES = 0.33), and sliding (*p* = 0.012, β = −6.00 s, ES = 0.24). This indicated that increasing age was associated with greater chasing games and rough and tumble play, whilst decreasing age was associated with greater sliding. The covariate ‘TGMD’ was associated with climbing/hanging (*p* = 0.043, β = 2.02 s, ES = 0.16), indicating that a greater TGMD score was associated with greater time spent climbing/hanging. The covariate ‘BMI’ was associated with standing (*p* = 0.008, β = −43.84 s, ES = 0.26), suggesting that increasing BMI was associated with decreased time standing.

## 4. Discussion

The results of this study highlight that children used a variety of equipment across the different playgrounds to engage in movement behaviour and, whilst no statistically significant differences were found for equipment dwell time, children spent more time interacting with the climbing equipment than with any other type. We hypothesise that this may be due to climbing equipment (such as climbing frames, climbing walls and bridges) often being some of the largest pieces of equipment on a playground. We acknowledge assertions regarding climbing equipment are based on an experience-based hypothetical explanation. Consequently, climbing equipment can offer space for multiple children to play at one time, resulting in dwell time accumulating more quickly. Likewise, the play value offered by these pieces of equipment is often more multifaceted than other pieces of equipment, therefore children are engaged for longer periods of time [46,47]. For instance, a climbing frame is likely to have multiple entry/exit points and routes to navigate through the piece of equipment, which require different skills to overcome. In contrast, a roundabout has fewer opportunities for engagement and requires fewer skills to interact with. Previous research has shown that children like to be challenged, often in the form of risk, and this can lead to greater engagement [48]. Equipment such as roundabouts may not offer sufficient challenges to engage children for longer periods of time, therefore resulting in lower levels of interaction.

Swing equipment and sand equipment were the second and third types of equipment most frequently interacted with across the three playgrounds. There were differences between gender as females spent notably more time on the swing equipment and males on the sand equipment. Whilst the dwell time on these pieces of equipment was not subject to statistical tests, the PA types most likely to occur on these pieces of equipment were found to be statistically significant. For instance, females spent more time performing swinging on the first playground, and males spent more time performing sand play on the second playground. These findings are similar to those in previous research, as Karsten (2003) [49] reported that females preferred swings and climbing frames on playgrounds, and Refshauge, Stigsdotter, and Petersen (2013) [50] reported that males preferred sand play. Differences in gender have frequently been reported across playground research [51].

Previous literature investigating ‘transitioning’ also reported similar findings to the current research. Foweather et al. [43] investigated transitioning in their study of school playgrounds and FMS. The authors concluded that transitioning was unable to support FMS development and instead, transitioning represented instances where children were “on the periphery of participating” in activities that could encourage FMS development. This lack of engagement may, in part, be due to limited ability (physical or social) or due to boredom. This was also the conclusion of Herrington et al. [52] who reported that children moved from one area to another in search of more engaging play and activities when bored. This is likewise supported by Adams et al. [36] who reported that on one of the playgrounds in their investigation, children were less engaged due to a lack of equipment and therefore created their own games, such as “chasey” for alternative entertainment. The results of the current study highlight that chasing games were one of the more frequently performed PA types. A key outcome from the statistical analysis was that age was a significant covariate for chasing games on the first and third playground (i.e., older children performed more chasing games than younger children), suggesting that older children may have been less engaged on these particular playgrounds.

One key outcome from the analysis was that age was a significant covariate for many of the different behaviours on the three playgrounds. On the first playground in this study, older children spent more time sliding compared to younger children, whilst the reverse was found on the second and third playgrounds. We theorise that this is because the structure and layout of the slides on the first playground appealed more to the older children, whilst the structure of the slides on the second and third playground appealed more to the younger children. One of the slides on the first playground was quite narrow and steep, and the older children were observed frequently using the narrow sides of the slide to assist climbing up the slide and then slide down it. In contrast, the slides on the second and third playground were quite wide with a small bump in the middle. The width made it more difficult to climb up, as both sides could not be held simultaneously, and the bump meant that running up the slide using momentum was less feasible. Though the first and second playgrounds did also contain enclosed tunnel slides, it is anecdotally noted that these were often used to sit and hide in rather than slide. These differences in behaviour may be explained by how children want to engage in risky behaviours (running up the slide), but often risk is reduced through design choices (wider slide with no sides to hold) due to fears surrounding injury [48,53].

Whilst age was a significant covariate for many of the different behaviours, TGMD score had less of an impact across the three playgrounds. TGMD score was associated with jumping/skipping and swinging on the first playground and climbing/hanging on the third playground. In each of these instances, a higher TGMD score was associated with greater time spent performing these activities. This implies that FMS ability enables children to engage with greater gross body movements on playgrounds. TGMD score also had an impact on some of the PA intensities observed across the three playgrounds. A greater TGMD score was associated with greater time performing limbs intensity PA on the second playground and moderate and slow/easy intensity PA on the third playground. These findings differ to those reported by Foweather et al. [43], who highlighted in their study that FMS had no association with PA intensity when they investigated school playgrounds and FMS ability. These differences may be due to dissimilarities in methods between Foweather et al. [43] and the current study. Foweather et al. [43] utilised school playgrounds and examined how children interacted with these spaces during break (recess) time, whilst the current study utilised public playgrounds during a non-school day. Considering that school playgrounds tend to have different equipment and layouts from public playgrounds, this may explain the differences in results. Likewise, break time during the school day is one of the few instances during an arguably sedentary day where children are encouraged to be active, so this may impact children’s behaviours. Therefore, the findings of Foweather et al. [43] may be more representative of behaviour on school playgrounds than public playgrounds.

In addition to the TGMD score, PA intensity was significantly associated with gender across the three playgrounds. Females were more likely to engage in high-intensity activity on the second playground, whilst males were more likely to engage in moderate-intensity activity on the second playground and stationary-intensity activity on the second and third playgrounds. Overall, the level of PA performed was less vigorous (i.e., limbs and stationary); therefore, these findings may be a representation of the overall PA level rather than a true difference between genders. Another explanation could be that activities that are likely to be performed at a lower level of intensity were performed more frequently on the second and third playgrounds. For example, activities such as sand play and lying down, which are performed at stationary/limbs intensity, were performed more on the second and third playgrounds, which may have influenced the results. Additionally, the fact that PA intensity was shown to be significantly associated with other variables, such as TDMG and age, suggests that there might be other factors that influence PA intensity on public playgrounds.

Research has reported that BMI can impact on the PA ability and PA engagement of children; therefore, we included it as a covariate in the statistical model [54]. However, findings of the present study showed that BMI had far fewer instances of impacting children’s behaviour and PA in comparison to age, gender, and FMS ability. BMI was only found to be statistically significant on the PA type standing, in which lower BMI was associated with increased time standing on the second and third playgrounds. These findings may be due to the influence of the equipment present on these playgrounds as opposed to BMI alone. Children were often observed waiting their turn for a certain piece of equipment, such as the zip wire and diggers (sand diggers), as they could only be interacted with by one child at a time. The limited impact of BMI on various aspects of children’s interactions with playgrounds suggests that BMI has less of an influence, particularly in comparison to factors such as age and gender.

Overall, children performed locomotor skills (e.g., run, walk), body control skills (e.g., climb, hang, push and pull), and sedentary activities (sit, lie down, stand) frequently on all three playgrounds. There was only one instance where children performed any object control skills (i.e., sand play); however, there were no gross motor object control skills performed (i.e., throwing and catching). This shows that public playgrounds are not made to support object control skills, as the equipment available does not create opportunities for children to perform these skills. These findings are likely to be a reflection of the lack of suitable equipment available on playgrounds that would encourage these skills to be performed, rather than a lack of ability or preference by children. The locomotor and body control skills that were performed were impacted primarily by age and gender, with FMS ability and BMI having a lesser impact. It should be noted that the statistical findings were not consistent across the three playgrounds, suggesting that there may be other factors that influence children’s behaviour. For example, social and cognitive ability, that impacts play and interactions, or perceptions and actualisation of affordances; however, it was beyond the scope of this study to investigate these factors.

### 4.1. Practical Implications

The findings of this study revealed that larger pieces of equipment, with multiple play opportunities, were more popular with children as such, future playgrounds should also seek to include larger equipment to encourage longer periods of play and PA. More consideration needs to be given to equipment designed for older children as the results of this study suggest that current play equipment provisions do not engage them as well as younger children. Suggesting concrete changes that could be made to playgrounds to facilitate object control skills as a consequence of this study is difficult as, to be feasible, such changes need to be co-created with architects, designers, urban planners and the users of playgrounds. Additional research conducted in this area to understand the wants and needs of older children/teenagers on playgrounds would also be useful in this regard. Additionally, there were few opportunities for children to perform object control skills. Therefore, it is recommended that designers of playgrounds be more innovative in creating play spaces that can encourage object control skills, either through novel, fixed equipment or through the provisions of loose, freely moveable equipment (such as bats and balls). However, it is acknowledged that providing loose equipment may be challenging due to theft concerns. Finally, the lack of consistent statistically significant findings across the three playgrounds suggests that there may be other factors that influence children’s PA and FMS on playgrounds. Therefore, more research needs to be performed that investigates these wider factors.

### 4.2. Strengths and Limitations

This study is one of the very few that have investigated how children interact with different types of playgrounds and play equipment in relation to children’s FMS ability, whilst using video footage to examine children’s behaviours. The use of video footage in the present study enables a thorough analysis of children’s playground behaviour, which has not been the case in prior work using direct observational methods. The present study utilised eight cameras and integrated methods from performance analysis, which provides a higher fidelity assessment of playground PA behaviour than has been conducted in previous research. Consequently, the results demonstrate the feasibility of assessing playground behaviour through multiple camera angles and multiple playgrounds in the UK context. Future studies are welcome to utilise the current study as a basis to inform future research/methods.

However, despite the use of a multi-camera setup, there were some instances where children went outside of the field of view, and the cameras could not capture what the children were doing, for example, if a child was inside a piece of equipment, such as a tunnel or a playhouse. Although participants were recruited from the communities in which the playgrounds were located, there is potential that the influence of novelty may have impacted the behaviours undertaken by the participants. Our decision to exclude the first 15 min of the observation period was taken to minimise the potential for novelty of experience to impact movement behaviour. Furthermore, while the sample size for this study could be considered modest, the sample utilised in the present study is comparable to prior work in the field using observational methodology [34,35,55].

Additionally, there are some variables that may have impacted the behaviours of the participants that the study could not account for, such as the impact of enjoyment, social ability and emotional ability. These factors may have impacted the ability of the children to engage in play and with the different pieces of equipment, which in turn may have impacted their behaviour and PA. Therefore, future research may wish to include these elements within their investigations.

## 5. Conclusions

This study examined how children interacted with different types of public playground and identify if their FMS ability had an impact on this interaction. Given the importance of FMS development to enable children to participate in PA and sports, understanding how playgrounds might implicitly support FMS development offers useful information for policy makers, planners, and the public. Using video-adapted OSRAC, the results of this study suggest that climbing equipment is the most frequently (average 1217.10 s) used type of equipment, and standing was the most frequently performed type of PA (average 377.60 s). Consequently, the most commonly performed intensity of PA was limbs (i.e., standing whilst moving arms and/or legs) (average 605.13 s) and moderate intensity activities (average 572.69 s). Furthermore, there were no instances of any throwing or catching performed on any of the three playgrounds. These results are notable because they suggest that public playgrounds do not facilitate more active types of PA (such as jumping) as well as they could, nor object control skills (in terms of throwing and catching). Therefore, designers of playgrounds may need to reevaluate what is on offer to children in playgrounds to encourage greater PA engagement. These results are also important because statistical analysis revealed that gender, age, BMI, and TGMD score were unable to consistently find differences for the various OSRAC variables across the three playgrounds. This suggests that the differences in equipment and layout across the three playgrounds may impact the behaviour and activities that children engage in.

## Figures and Tables

**Table 1:** The average time spent on different types of equipment.

Dwell Time on Equipment Types	All Three Playgrounds					
	Whole Sample		Males		Female	
	Average Seconds	Number of Participants	Average Seconds	Number of Participants	Average Seconds	Number of Participants
Activity trail equipment ^A^	214.90 ± 170.21	21	258.13 ± 210.94	8	182.77 ± 144.77	13
Climbing equipment ^B^	1217.10 ± 781.79	29	1075.30 ± 702.41	10	1278.37 ± 837.25	19
Sand equipment ^C^	746.70 ± 952.57	27	1241.30 ± 1124.41	10	419.71 ± 607.99	17
Seated equipment ^D^	728.59 ± 427.97	29	690.20 ± 433.30	10	746.42 ± 437.75	19
Swing equipment ^E^	828.71 ± 485.97	28	655.40 ± 481.52	10	875.68 ± 484.60	19
Other equipment ^F^	243.69 ± 190.76	29	205.90 ± 198.41	10	263.58 ± 188.98	19

A = 9 pieces of equipment, B = 9 pieces of equipment, C = 7 pieces of equipment, D = 8 pieces of equipment, E = 8 pieces of equipment, F = 9 pieces of equipment.

**Table 2:** The average time spent performing each type of activity on each playground.

Activity Type	All Three Playgrounds	Playground One ^a^						Playground Two ^b^						Playground Three ^c^					
	Whole Sample	Whole Sample		Males		Female		Whole Sample		Males		Female		Whole Sample		Males		Female	
	Average Seconds	Average Seconds	Number of Participants	Average Seconds	Number of Participants	Average seconds	Number of participants	Average Seconds	Number of Participants	Average Seconds	Number of Participants	Average Seconds	Number of Participants	Average Seconds	Number of Participants	Average Seconds	Number of Participants	Average Seconds	Number of Participants
Chasing games	118.19 ± 64.17	135.11 75.31	9	152.17 ± 67.80	6	101.00 ± 92.60	3	33.43 ± 21.14	7	28.50 ± 18.70	4	40.00 ± 6.51	3	172.00 ± 79.26	6	176.25 ± 77.26	4	163.50 ± 115.26	2
Climbing hanging	173.43 ± 150.70	160.55 ± 122.19	29	135.60 ± 64.52	10	173.68 ± 135.02	19	116.89 ± 119.21	27	114.56 ± 108.76	9	108.18 ± 123.73	17	246.50 ± 180.70	26	262.30 ± 236.41	10	236.63 ± 143.59	16
Crawling	12.11 ± 12.32	6.50 ± 6.54	12	4.25 ± 2.63	4	7.63 ± 7.74	8	26.50 ± 40.33	6	48.33 ± 51.29	3	4.00 ± 2.83	2	9.27 ± 5.04	11	11.20 ± 16.08	5	7.67 ± 3.72	6
Jump/skip	17.11 ± 19.43	17.67 ± 17.68	27	15.30 ± 11.55	10	19.06 ± 20.67	17	13.26 ± 17.59	26	24.50 ± 25.12	8	9.24 ± 10.85	17	20.39 ± 24.86	23	25.67 ± 16.08	9	17.00 ± 29.24	14
Lie down	46.57 ± 44.00	17.09 ± 18.26	11	23.00 ± 22.96	6	10.00 ± 7.71	5	88.00 ± 108.35	7	50.20 ± 67.63	5	182.50 ± 164.76	2	50.00 ± 59.43	10	39.33 ± 60.38	3	54.57 ± 63.25	7
Pull/push	97.16 ± 141.44	95.05 ± 97.47	21	149.86 ± 114.82	7	67.64 ± 78.14	14	81.19 ± 82.68	26	86.50 ± 86.37	8	78.50 ± 83.69	16	187.29 ± 254.11	17	347.67 ± 354.69	6	99.82 ± 128.70	11
Rough and tumble	46.24 ± 31.25	23.45 ± 27.86	11	22.14 ± 23.08	7	25.75 ± 38.86	4	27.67 ± 32.84	6	61.00 ± 43.84	2	11.00 ± 6.78	4	91.17 ± 66.57	6	73.25 ± 77.96	4	127.00 ± 8.49	2
Run	85.88 ± 67.83	74.41 ± 45.43	29	378.40 ± 220.32	10	73.84 ± 47.43	19	65.36 ± 47.86	28	98.13 ± 70.76	8	53.06 ± 27.37	18	120.77 ± 90.13	26	142.50 ± 113.66	10	107.19 ± 72.64	16
Sand play	260.38 ± 259.94	200.52 ± 210.01	14	378.40 ± 220.32	5	101.67 ± 128.96	9	140.00 ± 226.67	17	242.57 ± 331.86	7	56.56 ± 54.66	9	439.63 ± 335.15	19	576.67 ± 275.24	6	376.38 ± 350.99	13
Sit/squat	222.29 ± 153.87	197.79 ± 113.10	29	178.00 ± 122.11	10	208.21 ± 128.96	19	217.35 ± 150.22	28	156.00 ± 99.09	9	256.61 ± 161.79	18	254.96 ± 185.25	26	185.70 ± 204.63	10	298.25 ± 163.93	16
Slide	17.84 ± 15.79	18.14 ± 15.51	22	22.86 ± 15.81	7	15.93 ± 15.41	15	12.91 ± 8.60	22	9.57 ± 6.97	7	13.93 ± 9.14	14	27.08 ± 24.10	13	11.60 ± 8.14	5	36.75 ± 26.08	8
Stand	377.60 ± 195.23	348.38 ± 184.85	29	436.90 ± 227.88	10	301.79 ± 143.33	19	400.04 ± 204.72	28	409.33 ± 141.95	9	407.22 ± 233.04	18	386.04 ± 160.37	26	345.60 ± 156.65	10	411.31 ± 162.38	16
Swing	224.70 ± 165.35	298.10 ± 182.30	20	272.80 ± 179.04	5	306.53 ± 188.79	15	73.80 ± 83.88	5	132.50 ± 125.16	2	34.67 ± 22.30	3	190.71 ± 137.62	21	137.33 ± 85.89	6	198.81 ± 150.71	16
Walk	267.82 ± 151.05	261.28 ± 137.81	29	244.00 ± 165.04	10	270.37 ± 124.75	19	255.04 ± 135.21	28	213.00 ± 88.97	9	274.50 ± 154,69	18	288.88 ± 163.54	26	229.30 ± 103.94	10	326.13 ± 185.05	16

Total number of participants present at each playground: a = 29, b = 28, c = 26.

**Table 3:** The average time spent performing each physical activity intensity as measured by OSRAC.

Physical Activity Level	All Three Playgrounds	Playground One ^a^						Playground Two ^b^						Playground Three ^c^					
	Whole Sample	Whole Sample		Males		Female		Whole sample		Males		Female		Whole Sample		Males		Female	
	Average Seconds	Average Seconds	Number of Participants	Average Seconds	Number of Participants	Average Seconds	Number of Participants	Average Seconds	Number of Participants	Average Seconds	Number of Participants	Average Seconds	Number of Participants	Average Seconds	Number of Participants	Average Seconds	Number of Participants	Average Seconds	Number of Participants
Stationary	43.86 ± 50.97	11.38 ± 9.02	8	14.00 ± 12.12	3	9.80 ± 7.79	5	31.65 ± 48.91	17	58.14 ± 69.12	7	12.00 ± 11.96	9	44.67 ± 54.45	3	66.00 ± 56.57	2	2.00 ± 0.00	1
Limbs	605.13 ± 252.76	542.90 ± 177.99	29	586.00 ± 202.97	10	520.21 ± 164.64	19	627.54 ± 168.69	28	555.00 ± 105.04	9	685.06 ± 195.61	18	650.81 ± 270.43	26	524.70 ± 236.28	10	729.63 ± 266.76	16
Slow-easy	368.78 ± 217.16	607.90 ± 235.49	29	597.00 ± 127.60	10	613.63 ± 279.33	19	324.64 ± 168.69	28	360.00 ± 191.68	9	290.67 ± 144.77	18	800.54 ± 286.37	26	771.71 ± 341.06	10	818.56 ± 256.87	16
Moderate	572.69 ± 316.80	316.76 ± 205.89	29	378.20 ± 140.56	10	284.42 ± 229.92	19	282.54 ± 114.38	28	365.44 ± 129.41	9	237.83 ± 83.31	18	519.69 ± 215.85	26	586.80 ± 215.95	10	477.75 ± 211.65	16
Fast	26.31 ± 26.72	44.05 ± 70.28	22	58.70 ± 94.54	10	31.83 ± 44.25	12	29.95 ± 26.42	22	41.18 ± 29.71	7	19.86 ± 20.21	14	58.24 ± 59.29	21	64.88 ± 73.8	8	54.15 ± 51.33	13

Total number of participants present at each playground: a = 29, b = 28, c = 26. Stationary movements refer to instances where a participant is motionless, e.g., lying or sitting. Limbs refers to instances where a participant is stationary but there is movement of the trunk, arms, or legs, e.g., sitting whilst swinging legs. Slow/easy refers to instances where a participant is taking part in movements at a slow and easy pace, e.g., slow crawling or walking. Moderate movements refer to movements performed at a moderate pace, e.g., walking at a brisk pace or climbing on monkey bars. Fast movements refer to movements performed at a fast pace such as running or repeated galloping.

## Data Availability

The data that support the findings of this study are available from the corresponding author upon reasonable request. The data are not publicly available due to privacy and ethical restrictions.

## Abbreviations

The following abbreviations are used in this manuscript:

FMS	Fundamental movement skills
PA	Physical activity
OSRAC	Observational System for Recording Physical Activity in Children
